# The partnership for influenza vaccine introduction (PIVI): Supporting influenza vaccine program development in low and middle-income countries through public-private partnerships

**DOI:** 10.1016/j.vaccine.2019.06.049

**Published:** 2019-08-14

**Authors:** Joseph S. Bresee, Kathryn E. Lafond, Margaret McCarron, Eduardo Azziz-Baumgartner, Susan Y. Chu, Malembe Ebama, Alan R. Hinman, Anonh Xeuatvongsa, Silvia Bino, Dominique Richardson, Rachael M. Porter, Ann Moen, Mark McKinlay, the PIVI Partners Group, Gayane Sahakyan, Sonam Wangchuk, Pan Ruowen, Zhang Yongchao, Cai Linlin, Coulibaly Daouda, Olgha Tarkhan-Mouravi, Phillip Gould, Phillip Muthoka, Gideon O. Emukule, Sandra S. Chaves, Marc-Alain Widdowson, Dinagul Otorbaeva, Viengphone Khanthamaly, Kristina Stavridis, Vladimir Mikic, Nicolae Furtuna, Dumitru Capmari, Burmaa Alexander, Erica Dueger, Mirkhamudin Kamolzoda, Joshua Mott, Afif Bin Salah, Marie Mazur, Alba Maria Ropero Alvarez, Sonja J. Olsen, Sara Mirza, Carmen Sofia Arriola, Jane Seward, Samantha Kluglein, Amanda F. Bolster, Nguyen Minh Hang, Jeffrey W. McFarland, Nga Ha Thu, Thoa Thi Minh Nguyen

**Affiliations:** f Ministry of Health, Yerevan, Armenia; g Royal Centre for Disease Control, Ministry of Health, Bhutan; h Hualan Biological Bacterin Co., Xinxiang, China; i Ministry of Health, Abidjan, Cote D’Ivoire; j Ministry of Health, Tblisi, Georgia; k Southeastern Asia Regional Office, WHO, New Delhi, India; l Ministry of Health, Nairobi, Kenya; m US CDC, Nairobi, Kenya; n Ministry of Health, Bishkek, Kyrgyzstan; o US Embassy, Vientiane, Laos; p Institute of Public Health, Skopje, Republic of North Macedonia; q Ministry of Health, Chisinau, Moldavia; r National Center for Communicable Diseases, Ministry of Health, Ulan Bataar, Mongolia; s Western Pacific Regional Office, World Health Organization, Manila, Philippines; t Ministry of Health and Social Protection of the Population, Dushanbe, Tajikistan; u Thai-US Collaboration, US CDC, Bangkok, Thailand; v Institut Pasteur, University of Tunis Al Manar, Tunis, Tunisia; w Seqirus, Summit, NJ, USA; x Panamerican Health Organization, Washington, DC, USA; y Centers for Disease Control and Prevention, Atlanta, GA, USA; z Task Force for Global Health, Atlanta, USA; aa General Department of Preventive Medicine, Ministry of Health of Vietnam, Hanoi, Viet Nam; ab US CDC-Vietnam, Hanoi, Viet Nam; a Influenza Division, National Center for Immunizations and Respiratory Diseases, Centers for Disease Control and Prevention, Atlanta, GA, USA; b Center for Vaccine Equity, Task Force for Global Health, Atlanta, GA, USA; c Global Immunization Division, Center for Global Health, Centers for Disease Control and Prevention, Atlanta, GA, USA; d Ministry of Health, Lao Peoples Democratic Republic, Vientiane, Laos; e Ministry of Health, Tirana, Albania

**Keywords:** Influenza, Influenza vaccine, low and middle-income countries, Vaccination programs, Vaccine policy, NITAG

## Abstract

Influenza vaccination remains the most effective tool for reducing seasonal influenza disease burden. Few Low and Middle-Income Countries (LMICs) have robust, sustainable annual influenza national vaccination programs. The Partnership for Influenza Vaccine Introduction (PIVI) was developed as a public-private partnership to support LMICs to develop and sustain national vaccination programs through time-limited vaccine donations and technical support. We review the first 5 years of experience with PIVI, including the concept, country progress toward sustainability, and lesson learned. Between 2013 and 2018, PIVI worked with Ministries of Health in 17 countries. Eight countries have received donated vaccines and technical support; of these, two have transitioned to sustained national support of influenza vaccination and six are increasing national support of the vaccine programs towards full transition to local vaccine program support by 2023. Nine additional countries have received technical support for building the evidence base for national policy development and/or program evaluation. PIVI has resulted in increased use of vaccines in partner countries, and early countries have demonstrated progress towards sustainability, suggesting that a model of vaccine and technical support can work in LMICs. PIVI expects to add new country partners as current countries transition to self-reliance.

## 1 Introduction

Each year, influenza results in the deaths of an estimated 290,000 to 650,000 people worldwide [1]. Influenza vaccines remain the best method of reducing influenza disease burden. While influenza vaccination programs have been conducted in high-income countries for many decades [2], influenza vaccines remain underused, especially in many LMICS where rates of influenza-associated mortality and hospitalizations are highest [1], [3], [4], [5]. In 2014, only 24% of low and low-middle income countries (LMICs) reported policies for use of influenza vaccine compared with 94% of high-income countries; and even fewer LMICs had nationally funded programs [2]. Recent surveys of manufacturers indicated that only 5% of influenza vaccines produced during 2004–2013 were distributed to countries in Africa, Asia and the Middle East, which comprise 47% of the global population [6], [7]. The reasons for low influenza vaccine coverage in LMICs are many, but include lack of national government demand because of poor recognition of disease burden and value, cost and complexity of influenza vaccination programs, and competing priorities for scarce health funds [6], [8], Recognizing the need to expand global use of seasonal influenza vaccination, in 2012 World Health Organization’s (WHO) Strategic Advisory Group of Experts (SAGE) updated its previous influenza vaccination for health workers (HW) and certain groups at high-risk for influenza complications [9]. The underuse of influenza vaccines in many countries represents a significant missed opportunity for disease prevention. Importantly, the absence of a seasonal vaccination program also reduces a country’s capacity to respond to pandemic influenza with vaccine when needed [10], [11], [12], [13].

The Partnership for Influenza Vaccine Introduction (PIVI) was founded in 2013 to support the development of sustainable seasonal influenza vaccination programs and pandemic vaccine preparedness in LMICs through time-limited provision of influenza vaccines, supplies and technical support for policy development, planning, implementation and program evaluation. PIVI grew from two initial vaccine donation projects in Lao Peoples Democratic Republic and Nicaragua [14], [15], [16], in which Ministries of Health used donated influenza vaccines to initiate or expand national seasonal vaccination programs. Using the lessons learned from these early collaborations, PIVI was established as a mechanism to reduce annual influenza-associated disease and enhance national pandemic preparedness through catalyzing the growth of seasonal influenza vaccination programs in LMICs. We review here the first 5 years of the program, documenting progress, successes, obstacles and lessons learned.

## 2 Program overview and methods

### 2.1 Program overview

PIVI is a unique partnership of three types of organizations: (1) national Ministries of Health, which develop influenza vaccine policies and are responsible for program implementation and evaluation; (2) industry partners that support the provision of appropriate vaccines, shipping and supplies to the program; and (3) technical partners and collaborators (e.g., CDC, WHO) that provide assistance to the Ministries of Health in planning the programs, supporting national evidence-based policy development, and program evaluations.

PIVI is based at the Task Force for Global Health in Atlanta, USA, which provides overall coordination and strategic direction for the partnership, in close coordination with the U.S. Centers for Disease Control and Prevention (CDC), which is also the principal technical partner and funder. The goals of the program are to create sustainable routine seasonal influenza vaccination programs, and to build the immunization infrastructure capacity to ensure timely and effective vaccine delivery during pandemics and epidemics. PIVI’s work builds on ongoing collaborations between Ministries of Health and CDC’s Influenza Division and supports WHO’s programs that enhance influenza surveillance, laboratory and response capacities, and support vaccine policy development, program planning and evaluation activities.

### 2.2 PIVI approach

**Fig. 1:**
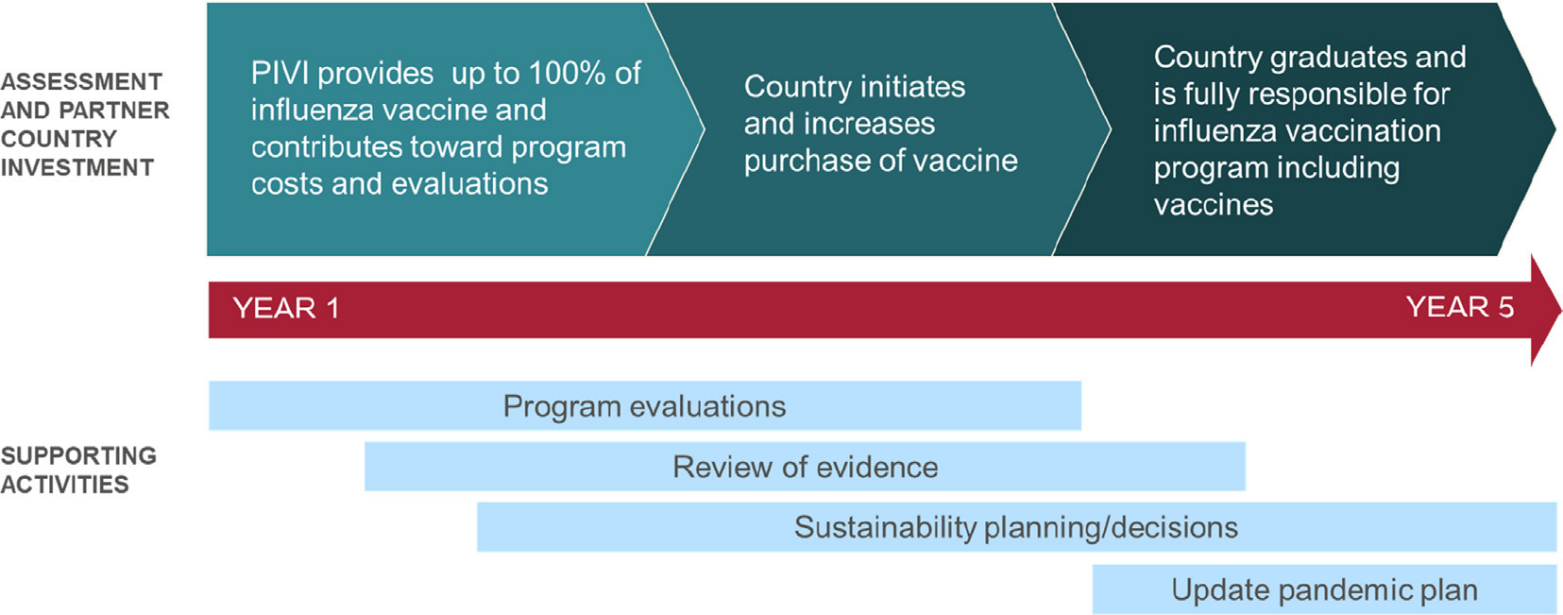
Paradigm for steps towards building sustainable influenza vaccination programs in PIVI partner country.

PIVI serves as a catalyst for countries to generate data and gain experience to support the development of decisions concerning and programs to carry out sustainable influenza vaccination programs. PIVI does this primarily through time-limited provision of donated or low-cost vaccines and/or technical assistance to partner countries. Countries receiving vaccine develop a multi-year plan (based on a 5-year template) for vaccine introduction in target risk groups, focusing on risk groups identified by WHO SAGE and the partner country. Subsequently, in these countries, PIVI provides vaccines that are donated by or purchased at reduced cost from manufacturers or procured from UNICEF during the first 1–3 years of the multi-year plan. After this initial period, the country progressively increases its financial contribution to vaccine purchase and program implementation (Fig. 1). At the end of the multi-year engagement, the country transitions from PIVI support by assuming full financial support of its national program.

A subset of PIVI partner countries receive only technical support rather than donated vaccine doses and supplies (Table 1). These “technical support-only” countries are of two types: (1) countries that will eventually receive PIVI-provided vaccine but request technical support for 1–2 years to plan and prepare for vaccine introduction, and (2) countries that have existing seasonal influenza vaccination programs but are interested in evaluating and improving the programs.

**Table 1:** Partner countries, by year of joining PIVI, and activities undertaken.

Year of PIVI engagement	Country^1^	Vaccine target group (planned)^2^					Initial year received donated vaccine (planned)	Technical support received (planned)						Status as of January 2019
		HW	PW	CD	OA	C		VE	AEFI	Econ	NITAG	PE	KAPP	
2013	Lao PDR^3^	X	X		X		2012	X	X	X	X	(X)		Engaged – expected Transition 2019
	Nicaragua		X				2013	X	X				X	Transitioned^4^ 2014
2014	Morocco			X			2014			(X)		X	X	Transitioned 2014
2015	Moldova	X		X			2015				X^5^		X	Active – exp. Transition 2021
	Armenia	X		X			2015			(X)	X		(X)	Active – exp. Transition 2021
2016	Mongolia	X	X			X	2016		X	X	X		X	Active – exp. Transition 2021
	Albania	X	X	X	X		2016			X	(X)		X	Active – exp. Transition 2021
2017	Kyrgyzstan	X	X	X			2017						X	Active – exp. Transition 2022
	Vietnam	X					(2019)				X	(X)	X	Active – exp. Transition 2023
	Uganda	X											X	Active
	Kenya					X	(2020)			(X)		(X)	X	Active
	Georgia	X	X							(X)	(X)^5^		X	Active
	Cote D’Ivoire	X				X	(2019)			(X)	X		X	Active
2018	Tajikistan	X	X				(2019)						(X)	Active
	Bhutan	X	X				(2019)			(X)		(X)		Active
	Rep. North Macedonia	X		X			(2019)				(X)		X	Active
	Tunisia	X		X									(X)	Active

Abbreviations: HW – Health Worker; PW – Pregnant Women; CD – Persons with chronic diseases; OA – Older adults (either >60 yrs or >65 yrs, depending on country); C – Children (either 6mo – 23 mo. or 6 mo. – 5 yrs. (depending on country); VE – Vaccine Effectiveness; AEFI – Adverse Events Following Immunization; Econ – economic activities, including estimating cost of vaccination program, costs of disease, or cost-effectiveness evaluations; NITAG – National Immunization Technical Advisory Committee; PE – program evaluation, such as influenza post-introduction evaluations or vaccine demonstration projects; KAPP – Knowledge, Attitudes, Perceptions and Practices survey.

1 Partner countries were either low, low-middle, or upper-middle income countries at the time of PIVI engagement as of 2018. Low-income countries included: Tajikistan, Uganda; low-middle-income countries: Laos, Nicaragua, Morocco, Moldova, Mongolia, Vietnam, Kyrgyzstan, Kenya, Georgia, Cote D’Ivoire, Bhutan, Tunisia; Upper-middle income countries: Armenia, Albania, North Macedonia.

2 Planned activities are any that will be conducted in 2019 or 2020.

3 Lao PDR received donated vaccine (405,903 doses) starting in 2012 through a donation from Walgreens Company prior to the organization of PIVI.

4 Transitioned indicates that the country is continuing the vaccination program using national resources.

5 Representatives from Moldova and Georgia attended the NITAG training in Armenia.

### 2.3 Country engagement and selection

All countries are evaluated for PIVI partnership through a systematic process based on criteria designed to identify LMICs that are interested, ready and capable of developing an influenza vaccination program. Readiness criteria relate to the existence of high-quality influenza surveillance data, disease burden data, and a formal statement of interest from the representatives from Ministries of Health to initiate influenza vaccination in at least one SAGE target group in the country [9]. Countries’ capabilities are assessed based on delivery of other vaccines and absence of significant challenges to program sustainability (e.g. ongoing conflict in the country). Potentially eligible countries are identified based on publically available data (e.g. current vaccine program performance, other recent and ongoing vaccine introductions, influenza surveillance capacity, etc.) with final country selection based on a series of engagements and discussions with the country representatives and other key stakeholders, such as the WHO Regional and Country Offices.

Once engaged, each country selected to receive vaccine doses develops a multi-year plan for either vaccine introduction or for expansion of an existing program, and for sustaining the programs over time. This plan outlines the country’s vaccine and supply needs and desired types of technical assistance during a five-year timeframe. For each year of the plan, the country indicates its preferred vaccine product formulation and presentation, the number of doses required each year, and the delivery date requested for vaccine shipments. These choices are based on seasonality of influenza, national vaccine target groups, vaccine coverage goals, and locally available or acceptable vaccines. Countries are encouraged to prioritize a small number of target groups based on the SAGE recommendations, and to begin the program with relatively modest vaccine coverage goals and with an aim to increase coverage over time.

### 2.4 Technical support

All countries receive technical assistance based on a review of gaps in the evidence-base for influenza vaccination in the country and identified needs in planning or evaluating the vaccination program. This technical assistance is provided from CDC and other technical partners, and funding to support in-country activities. Technical assistance offered to a country is tailored to address data and program gaps expressed by local stakeholders and/or capacity improvement needs. To facilitate technical support, PIVI has developed a panel of generic tools that, alongside available WHO-developed tools [17], [18], are then modified for a specific country context (Table 1). The creation of tools was in part based on a 2017 partner country survey that collected information on key challenges to developing sustainable seasonal influenza vaccination programs.

## 3 Results

From 2013 to 2018, 17 countries became PIVI partners (Table 1). Eight countries have received donated vaccines and supplies, while 9 countries have received only technical assistance to date. Between one and four countries have been added to the partnership each year from 2013 to 2018. In 2018, 15 countries were actively engaged in the partnership. During the 6-year period, eight countries received a total of 3,671,078 vaccine doses from PIVI from four manufacturers (Seqirus, Hualan Bacterin, Green Cross, and Protein Sciences (now Sanofi Pasteur)). Among the 25 country-years of vaccine donations, the median number of doses per year received by a country was 92,500 (interquartile range: 55,760–153,000).

### 3.1 Program sustainability and country ownership

Each vaccine-receiving country developed multi-year plans that summarized the annual needs for vaccine donations, the plan for country purchase of vaccine, and the timing of technical activities to provide the evidence base for program sustainability and country investment. The countries 5-year targets for vaccine coverage among risk groups was between 18% and 74%, with lower targets (4–47%) in the early years. The higher target coverage in some countries reflected health workers as an early target, which tend to be the smallest target group among SAGE-recommended groups. All 17 countries either carried out or are planning vaccination of SAGE-recommended target groups. The more commonly targeted group for vaccination has been HW (14 countries), followed by pregnant women (9) and persons with chronic diseases (7). Technical or data needs varied and were specific to each country and include activities related to estimating disease or economic burden, expected impact of vaccination, review of evidence base for vaccination, programmatic information, and program evaluation projects.

**Fig. 2:**
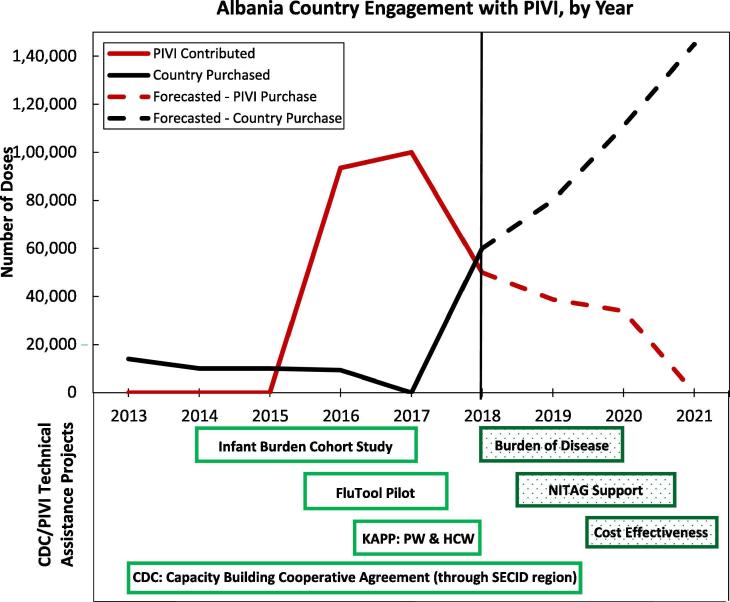
Example of country processes and progress in introducing or expanding influenza vaccination within the PIVI framework – Albania. FluTool- The WHO tool for estimating economic costs of introducing influenza vaccination; NITAG – National Immunization Technical Advisory Group. KAPP – Knowledge, Attitudes, Perceptions and Practice survey; PW – pregnant women; HCW – healthcare workers; SECID – South East European Center for Infectious Disease.

All countries that received vaccine also conducted activities to plan, evaluate and improve the vaccination program. One example of the combination of vaccine donations, local purchase of vaccine and technical collaborations underlying the multi-year plan is provided by Albania (Fig. 2). Albania has conducted a variety of evaluations and data collections to create an evidence base for influenza vaccination policy decisions, and has used these data to increase and stabilize country vaccine purchase over time.

Among the eight countries that have received donated vaccine since 2013, two have transitioned to country-funded vaccination programs (Table 1). Each of the 6 currently engaged countries began its own vaccine purchase during years 2–4 of the PIVI engagement, and has increased the number of vaccine doses purchased using government funds and decreased receipt of donated vaccines over time in accordance with their multiyear plan during this period. Countries that received donated vaccine purchased 43% more seasonal influenza vaccine in 2018 compared with their average purchases during the three years before PIVI engagement (445,000 doses vs. 310,000 doses, respectively). Like Albania, the other countries which have increased purchase have done so using Ministry of Health funds, based on building and communicating the evidence base for vaccine over time. Lao PDR is expected to fully sustain its vaccine program by 2020 based on its multi-year sustainability plan, while the remaining five are expected to transition to be fully sustainable by 2023. Five new partner countries will receive vaccine donations in 2019 (Bhutan, Tajikistan, Cote D'Ivoire, North Macedonia and Vietnam), and have multi-year plans in place that forecast transferring vaccine purchase to the national budget by 2024.

The nine countries that have received only technical support (no donated vaccine) have been engaged for fewer than two years, making it difficult to draw conclusions about the value of receipt of technical assistance alone in developing a sustainable vaccination program. However, early results are encouraging. Based on evidence and experience gained from a small demonstration project among health workers using a knowledge, attitudes, perception and practices (KAPP) survey and National Immunization Technical Advisory Committee (NITAG) workshops, in 2017, Vietnam developed a national policy targeting health workers and a multi-year plan for step-wise introduction of HW vaccination countrywide. In Kenya, after NITAG strengthening supported through a CDC cooperative agreement that preceded PIVI the recent KAPP surveys, the Ministry of Health developed a national policy to vaccinate young children and will conduct a sub-national demonstration project of vaccinating children 623 months in 2019 [19]. Similarly, Cote D’Ivoire received support for NITAG strengthening and KAPP surveys, which supported development of national recommendations for vaccination of HWs, and began a phased roll-out of vaccination in 2018. In the next year, five additional countries will receive donated vaccine (Vietnam, Bhutan, Cote D'Ivoire, Republic ofNorth Macedonia and Tajikistan), two will continue to receive only technical assistance towards expanding or enhancing the use of nationally purchased vaccine (Georgia and Tunisia), and two will be considered for future vaccine support based on the results of currently ongoing or planned evaluations through technical assistance (Uganda, and Kenya).

Partner countries identified several challenges to introducing, expanding or sustaining influenza vaccination programs (Table 2). Some of the obstacles were related to concerns about the safety and/or value of vaccination. These gaps were often addressed in the first year or two of the partnership by collecting new data using generic tools, or by using the influenza resource package created for NITAG use. Challenges related to lack of capacities, such those related to provision of well-informed policy decisions and management for vaccination, distribution planning or regulatory authority education were addressed either with targeted training and technical assistance opportunities (e.g. NITAG training), or through the actual vaccine delivery during annual campaigns (e.g. microplanning). Concerns about the paucity of available products and the cost of vaccines that are required to be administered annually will remain challenging as countries proceed towards transition to local support. The access to vaccines might further be threatened as producers focus increasingly on more expensive formulations (e.g. quadrivalent vaccines), and if newer products are not WHO-prequalified. Additional years of experience will be needed to identify solutions in all countries, and careful cost-effectiveness analyses will be helpful in making decisions for use of these vaccines.

**Table 2:** Challenges noted by partner country points of contact in developing sustained seasonal influenza vaccine programs.

Challenge type	Specific challenge
Lack of documentation of value of vaccination	Limited or uncertain disease burden / value of vaccination Limited data on risk groups Concerns about vaccine effectiveness in potential target groups
Vaccine safety	Concerns about use in pregnant women
Stakeholder/policy makers need education	Need for stakeholder communication materials Negative experience with 2009 pandemic vaccines
Unprepared regulatory landscape for influenza vaccines	Few or no influenza vaccines currently approved NRAs lack experience with influenza vaccines
Lack of national influenza vaccination policy	Inexperienced NITAGs Lack of sufficient local subject matter expertise
Poor program readiness	Lack of existing programs accessing SAGE-recommended target groups Lack of distribution plan Limited cold chain capacity to accommodate seasonal bolus of vaccine doses
Product issues	Concerns regarding short product expiry dates, especially in countries with year-round disease
Costs	Costs of vaccine Cost-effectiveness of vaccination programs

### 3.2 Technical and financial support provided

All 17 countries have received technical and/or financial support for program and/or policy development and evaluation (Table 1). KAPP surveys about influenza and vaccination have been conducted or are planned for 2019 in 16 countries with the first work occurring in Lao PDR [20]. These surveys focused on vaccine target groups, including health workers (12), pregnant women and their providers (12), persons with chronic disease (3) or caretakers and providers of health care for children (1). Data from the surveys have been used to inform appropriate target groups for vaccine, to plan communication and educational campaigns, and to evaluate the success of these efforts. Vaccine effectiveness evaluations have been conducted in two countries, both focused on the effect of vaccination of pregnant women on their pregnancy and birth outcomes. These evaluations in Lao PDR [15] and Nicaragua [16], [21] provided data to the national governments to understand the value of the programs and to inform policymakers of the effectiveness and safety of vaccine in these groups. While all countries are required to conduct surveillance for adverse events following immunization (AEFIs) in conjunction with the vaccination program, two (Mongolia and Lao PDR [14]) have conducted enhanced or active surveillance for AEFIs to produce data requested by manufacturers and national policymakers. Economic evaluations, including estimating the costs of influenza illness and the programmatic costs of influenza vaccination have been conducted or are planned in 9 countries, using the WHO tools for economic evaluations or other available instruments [17], [22], [23], [24]. Finally, a post-introduction evaluation was conducted in Morocco and are being planned in 4 countries [18].

Capacity-building workshops for country NITAGs have been conducted for 6 countries since June 2017 (Table 1). Training courses were conducted for general NITAG capacity building (2 courses) and for influenza working group development and general NITAG capacity building (3 courses). Three additional NITAG training courses are planned for 2019. The NITAG training curriculum was based partly on materials developed by WHO and the Supporting Independent Immunization and Vaccine Advisory Committees (SIVAC) [25], [26], and experts who worked as part of the SIVAC project were engaged in PIVI training. Follow-up technical support was provided to countries following the training courses. In addition, an Influenza Resource Package was developed to support NITAG training and subsequent discussions of national policy development. The influenza resource package included a systematic literature review of papers relevant to national discussions about disease epidemiology and disease burden, vaccine effectiveness and safety, and program implementation issues. The Influenza Resource Package was modified to include data relevant to the country and region conducting the NITAGs, and will be made available to any interested country through PIVI participation and/or the WHO NITAG Resource Center.

## 4 Discussion

During the first six years of PIVI, the program has supported 17 LMICs to initiate or expand influenza vaccination programs targeting SAGE-recommended target populations and to gather data and gain experience needed to make decisions on the value of sustaining influenza vaccination programs using national resources. PIVI has produced early evidence that a paradigm of provision of technical support and making vaccines available can result in establishing or expanding a seasonal influenza vaccine program and sustained government funding over time. Six countries that have received vaccine and technical support for at least 3 years have steadily increased their financial support of the program and are expected to be fully self-sustaining within the next 1 to 3 years. In addition, two countries that received PIVI support early in the program have transitioned to conducting annual vaccination campaigns of target groups using only national resources. Additional countries that have recently joined PIVI have received only technical support – either as a preparation for future vaccine donation or as a tool to increase efficiency or impact of their current influenza vaccination program. Experience thus far indicates that provision of expertise to address critical data gaps identified by local stakeholders has resulted in creation of national vaccine policies and plans to initiate national vaccination programs. Finally, countries participating in PIVI have developed processes and capabilities for vaccine delivery that will benefit the country in the timely and effective use of pandemic and non-influenza epidemic vaccines when needed. We note, however, that these results are early and interim, and that a better assessment of the value of this model will take several years.

A central tenet of the need for seasonal influenza vaccination programs is the value of annual programs to create the national and global capacities to deploy pandemic or non-influenza epidemic vaccines quickly and effectively when needed [8], [9], [27], [28], [29]. Countries with seasonal influenza vaccination programs in 2009 deployed vaccine more effectively than those without such programs in place [13]. Reviews conducted after the pandemic also indicated that countries that deployed vaccine late or not at all lacked critical capacities required for vaccination program, such as capable regulatory systems, national policies for use of vaccine, and vaccine deployment plans [12], [13], [30]. In addition, a person’s acceptance of pandemic vaccine was associated with prior receipt of seasonal vaccine [31], [32], [33]. As a result, some countries with no or poor seasonal vaccination programs encountered low acceptance of the vaccine, resulting in low coverage that limited the impact of the vaccination programs [30]. Currently, few LMICs have robust routine influenza vaccination programs, outside of the Americas [2], [34], [35], [36], [37]. While substantial and successful work has been carried out in LMICs during the past 30 years to build childhood immunization programs, countries’ abilities to vaccinate older children and adults remains limited. Because vaccines that target pandemic influenza or certain epidemics, such as Ebola virus, will likely target adult populations, especially health workers, seasonal influenza vaccination programs can help build these capabilities. In addition, because seasonal influenza vaccination programs are carried out annually, countries that conduct annual campaigns using seasonal influenza vaccination have repeated practice procuring and deploying these vaccines. Because of the link between pandemic and seasonal preparedness, PIVI partner countries will work to update their pandemic vaccine plans in 2019–2020, building on the capacities developed for seasonal vaccination. From a global perspective, increased demand from LMICs might help drive increased vaccine production capacity from multinational and emerging suppliers of vaccine [38]. This can both help ensure that sufficient production capacity is available during the next pandemic and lower prices per dose for seasonal vaccine.

We have learned several lessons during the initial period of PIVI. First, we have found that many LMICs are interested in initiating and expanding their seasonal influenza vaccination program, despite competing priorities for health funds in the country. Slow development of programs in many countries is based to uncertainty of the value and feasibility of influenza vaccination. By lowering the country risk to initiate or expand vaccine programs by sharing program costs with PIVI early in program development, and by facilitating technical assistance to address critical knowledge and capacity gaps, countries have been willing to institute seasonal influenza vaccination while they gather data to understand the potential costs and value of the program. In addition, creating a multi-year plan that contains modest targets for vaccine coverage of risk groups (so requires a modest number of doses) with gradual increases somewhat reduces concerns of affordability and sustainability. Second, all countries requested technical support to plan, conduct, or evaluate their program. By having available generic protocols and data collection tools for the various types of technical assistance, PIVI can be efficient in providing this support. Close coordination and communication with WHO and WHO Regional Offices and other experts has been important for development and testing of technical support tools [17], [18], and for NITAG strengthening [39]. Third, the work with the first group of 17 countries has been built on years of effort to build surveillance and laboratory capacity in the countries which have produced the data for program design and to engage country policy-makers [40], [41], [42]. Continued support of influenza surveillance globally, especially in low-income countries should remain a priority [8]. Fourth, because each country has unique history with influenza vaccines and capacities, the timeline to sustainability will vary. Nicaragua and Morocco, for instance, had very targeted goals to expand existing use of vaccine to new areas or risk groups. Their work built on existing systems and policies, and resulted in a transition from PIVI donations after only 2 years. Countries just starting programs or with less experience with influenza vaccines will likely need support for a longer period. Finally, regional support through WHO Regional Offices or existing regional structures (e.g. South East European Center of Infectious Diseases (SECID)) can add efficiency to the process of program building and opportunities for peer support. Regional collaboration may also be a future mechanism for lower cost vaccine purchased through pooled procurement in the future [34].

It is worth noting how this influenza vaccine donation program, or any influenza vaccine donation, meets or fails to meet requirements established for vaccine donation by WHO and UNICEF [43]. Five minimum requirements are defined in the guidance to ensure best practices are maintained. Three of the requirements (programmatic and epidemiologic suitability to the country, the presence of fully informed health officials, and that the vaccines are locally approved by the regulatory authorities) were addressed in all participating countries. The requirement for sustainability of the program after the donation is a key tenet of the program and participation requires plans for sustaining the vaccination programs. While the vaccine and importation characteristics of PIVI donations satisfy the WHO-UNICEF requirements, donated influenza vaccines do not meet the criterion that donated vaccines have 12 months of remaining shelf life. Indeed, the expiry dates on influenza vaccines are designed to be less than 12 months to ensure that vaccines are used or destroyed before the distribution of the next formulation. As a result, influenza vaccine doses cannot meet this criterion. Even so, because the vaccines are given in planned campaigns ahead of vaccine expiry, this has not been raised as a problem among participating countries. Influenza vaccines have unique characteristics for which amended guidance from WHO and UNICEF might be of value.

While the first 17 PIVI partner countries have been a source of lessons learned, expansion to the next group of countries, especially lower income countries, may require different approaches. Lessons from Gavi and other recent vaccine introductions will be helpful to develop support for these countries [44]. With the recent Gavi decision to invest in evaluating seasonal influenza vaccination for health workers, opportunities to increase experience and develop best practices in the lowest income countries will increase in the coming years, augmenting PIVI’s knowledge base [45]. LICs might benefit from a modest annual purchase of vaccine, perhaps targeting only health workers, rather than a large program that could strain national resources. For instance, in Vietnam, the estimated size of the targeted health worker population is approximately 300,000, compared with approximately 1.6–1.7 million pregnant women and 7.5 million children under 5 years. In this setting, limited vaccination of this single target group to enhance vaccine regulation, policy making, deployment plans, and vaccine acceptance towards pandemic and epidemic readiness, rather than focus primarily on disease reduction, might be acceptable and affordable. Evaluating the costs and benefits of this model will be an important next step.

PIVI uniquely fills critical gaps in global influenza prevention and control and pandemic preparedness. Seasonal influenza vaccination remains the best current tool to reduce the 290,000–650,000 influenza-associated deaths each year in the world [1], as well as a tool to build required national and international capacities for pandemic and epidemic vaccine delivery. PIVI is based on the concept that solutions to global influenza disease prevention and control will require participation of both public and private entities. Key challenges remain including availability of affordable vaccines, development of more effective, easier to deliver vaccines, and strengthening the evidence base relevant to specific target populations in LMICs. While progress in these areas is being made, further efforts are needed to realize the benefits of vaccines for seasonal and pandemic disease reduction. We think that PIVI is a promising model for expanding access to influenza vaccines, and can complement ongoing efforts of global partners, industry and national governments towards this goal.

## Funding

This work was supported by Centers for Disease Control and Prevention, Atlanta, GA, USA [CDC-RFA-IP16-1607] and the Bill & Melinda Gates Foundation, Seattle, WA, Seattle, WA, USA [OPP1088249]

## Declaration of Competing Interest

The authors declare that they have no known competing financial interests or personal relationships that could have appeared to influence the work reported in this paper.
